# 6 February 2023, orthopedic experience in Kahramanmaraş earthquake and surgical decision in patients with crush syndrome

**DOI:** 10.1186/s13018-023-04001-2

**Published:** 2023-07-27

**Authors:** Bugra Kundakci, Akif Mirioglu, Mustafa Tekin, Melih Bagir, Omer Sunkar Bicer, Yusuf Kemal Arslan, Cenk Ozkan, Hilmi Serdar Ozbarlas

**Affiliations:** 1 Department of Orthopaedics and Traumatology, Cukurova University Faculty of Medicine, Adana, Turkey; 2 Department of Biostatistics, Cukurova University Faculty of Medicine, Adana, Turkey

**Keywords:** Crush syndrome, Earthquakes, Amputation, Fasciotomy, Compartment syndrome

## Abstract

**Background:**

The decision of fasciotomy or amputation in crush syndrome is controversial and challenging for surgeons. We aimed to share our experiences after the Kahramanmaraş earthquake, to predict the severity of crush syndrome and mortality, and to guide the surgical decision.

**Methods:**

The clinical data of patients during their first week of hospitalization were analyzed retrospectively. Totally, 233 crush syndrome patients were included. Demographic data, physical and laboratory findings, surgical treatments, and outcomes were recorded.

**Results:**

The mean time under the rubble was 41.89 ± 29.75 h. Fasciotomy and amputation were performed in 41 (17.6%) and 72 (30.9%) patients. One hundred and two patients (56.7%) underwent hemodialysis. Fifteen patients (6.4%) died. Lower extremity injury, abdominal trauma, and thoracic trauma were associated with mortality. Mortality was significantly increased in patients with thigh injuries (*p* = 0.028). The mean peak CK concentration was 69.817.69 ± 134.812.04 U/L. Peak CK concentration increased substantially with amputation (*p* = 0.002), lower limb injury (*p* < 0.001), abdominal trauma (*p* = 0.011), and thoracic trauma (*p* = 0.048).

**Conclusions:**

Thigh injury is associated with the severity of crush syndrome and mortality. Late fasciotomy should not be preferred in crush syndrome. Amputation is life-saving, especially in desperate lower extremity injuries.

On 6 February 2023, a 7.8 magnitude on the Richter scale a catastrophic earthquake struck at 04:17 local time with its epicenter located in the Pazarcık district in Kahramanmaraş province, Turkiye. This is the highest magnitude earthquake recorded in Turkiye since the 1939 Erzincan earthquake, and over 9000 aftershocks followed it. The earthquakes directly affected 11 provinces within Turkiye. Approximately, 15 million people live in these provinces, including over 1.7 million Syrian refugees and around 4.6 million children. More than 520 000 individual units in 164 321 buildings either collapsed or were heavily damaged in Turkiye, including at least 15 hospitals. It has been reported that approximately 50 million people died, and more than 100 million people were injured in Turkiye [1].

Most of these injured patients were trapped under the rubble and suffered varying degrees of limb crush injury. The severity of the condition is related to the magnitude and duration of the compressing force, and the mass of muscle affected [2]. The association between crush injuries and kidney failure was first documented by Bywaters and Beall in 1941, who had extensive exposure to patients crushed by falling debris during the bombing of London [3].

Crush injury is caused by continuous and prolonged pressure on the limbs. Clinically, It may present with diffuse swelling, erythema, blisters, purpura, open fractures, ischemia, and tissue necrosis. In addition to the management of expected orthopedic and vascular problems, it should be kept in mind that major systemic manifestations may occur due to reperfusion after prolonged hypoperfusion [4].

Crush syndrome is the systemic manifestation of rhabdomyolysis resulting from pressure or crushing. Crush syndrome is characterized by shock, hyperkalemia, hypocalcemia, metabolic acidosis, renal failure, and often compartment syndrome. Renal failure is multifactorial and results in combinations of multiple causes, including hypovolemia and the release of large quantities of nephrotoxic substances from injured muscle cells. Electrolyte abnormalities such as hyperkalemia can cause negative inotropy and potentially fatal arrhythmias [5]. Early fluid resuscitation, within the first six hours, is essential [6].

The objectives of surgical treatment of crush injuries include saving lives and restoring or preserving functions. Fasciotomy is effective in reducing intra-compartmental pressure and treating compartment syndrome. But it is often the cause of complications such as crush syndrome due to revascularization, infection, and sepsis, and threatens life. In patients with crush injuries, extensive muscle necrosis is a potential source of myoglobin and potassium for circulation. Amputation of desperate limbs removes this source and may save lives [7].

Ensuring error-free triage is critical in saving more patients’ lives and providing the right patient with immediate emergency treatment in such mass disasters where there are many applications in emergency services. However, determining the priority is challenging for clinicians since there is no applicable guideline as far as we know. The time trapped under the rubble, concomitant injuries, and affected part of the extremity may affect the patient's clinical status and decision. In this study, we aimed to share our experiences after the Kahramanmaraş earthquake to predict the risk of crush syndrome and mortality according to the affected limbs.

## Methods

One thousand ninety-six earthquake victims, mainly from Hatay, were admitted to our hospital. Four hundred patients were examined, treated, and followed up with the diagnosis of fracture, crush injury, soft tissue laceration, and traumatic amputation.

**Fig. 1:**
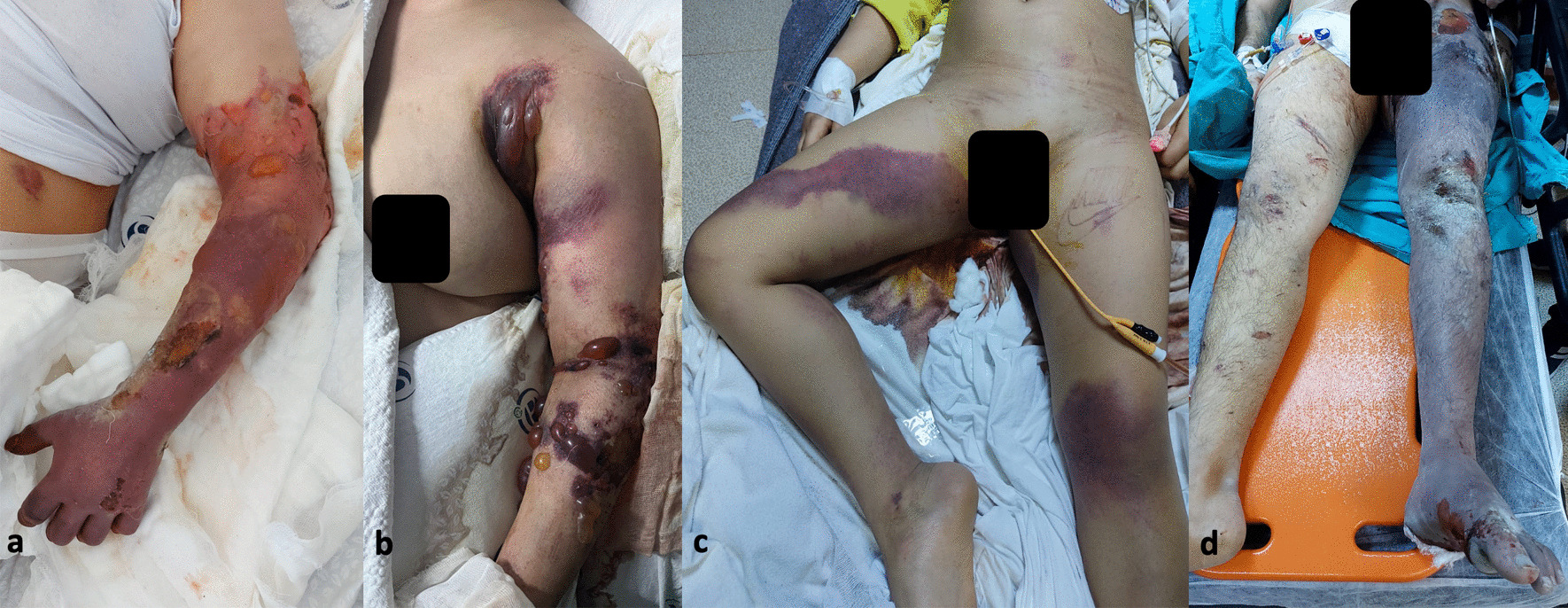
Various degrees of crush injuries. **a** tissue necrosis of the hand and forearm and bullae in the arm. **b** hemorrhagic bullae on the left arm and forearm. **c** Brand logo mark due to excessive swelling on left thigh **d** Cyanosis and necrosis of the left lower limb

After obtaining approval from the local ethics committee, patient files were reviewed retrospectively from the hospital database. Age, gender, nationality, admission date, time under the rubble, surgical procedures, surgery time, abdominal injuries, thoracic injuries, creatinine kinase (CK) concentration, hemodialysis requirement, crush injuries in the arms, forearms, hands, thighs, legs, and feet were recorded. Crush injury was diagnosed based on a history of limb compression and manifested clinically as diffuse swelling and tension, paralysis, pulselessness, erythema, bulla formation, cyanosis, ischemia, necrosis, and open fractures of limbs (Fig. 1).

We aimed to determine the development of crush syndrome, dialysis requirement, and CK concentration according to the crushed limb parts. Patients admitted to our hospital in the first week after the disaster were included in the study. Patients who admitted after the 7th day of the disaster and had a hospitalization period of less than a day were excluded from the study. Among the patients, those with a CK concentration below 1000 U/L, which is an indicator of rhabdomyolysis, were excluded from the study [8, 9]. For the final evaluation, 233 patients with crush syndrome were included. In this patient group, the effects of trapped time, affected limb parts, surgical procedures on CK concentration, dialysis requirement, and mortality were examined.

When the thighs were crushed, the legs and feet were affected secondary to ischemia. This led us to the misconception that the crush syndrome clinic was severe in foot and leg injuries. For this reason, patients with lower limb injuries with crush syndrome were divided into four groups according to the parts of the affected limb. The first group is those who do not have crush injuries in their lower extremities. The second group is those with lower limb crush injuries other than the thigh, the third group is unilateral thigh or unilateral thigh plus other parts, and the fourth group is bilateral thigh or bilateral thigh plus other limb parts. The mortality rates of the groups were compared.

Our hospital was damaged after the earthquakes, and the main buildings were evacuated on 21.02.2023. On the 15th day after the disaster, the patients were transferred to other health institutions. We could not do long-term follow-ups of the patients.

### Statistical analysis

While continuous variables were summed up as mean, standard deviation, median, and quartiles (Q1–Q3), categorical variables were expressed as numbers and percentages. To compare categorical variables between the groups, the chi-square test was performed. The Kolmogorov–Smirnov test was used to confirm the normality of the distribution for continuous variables. Mann–Whitney U test was used to compare continuous variables between two groups. To compare more than two groups, the Kruskal–Wallis test was employed. IBM SPSS 20.0 (Armonk, New York, IBM Corp.) was used for all statistical analyses. A value of *p* < 0.05 was accepted as statistically significant for all tests.

## Results

Ninety-six limb amputations were performed on 77 patients (Table 1). Excluding ray and phalanx amputations, 89 amputations were performed in 70 patients. In addition, stump revision surgeries were performed in seven of 17 patients referred to our clinic after amputation in other health institutions. The mean surgical time for amputations was 105.71 ± 57.65 h. Crush syndrome developed in 72 patients who underwent amputation. Crush syndrome did not exist in two patients with severe crush injury who underwent transhumeral and transfemoral amputation at the 8th and 16th h, respectively.

**Table 1:** Amputation levels

Amputation levels	Number of amputations
Shoulder disarticulation	1
Transhumeral amputation	8
Elbow disarticulation	1
Forearm amputation	7
Metacarpal-phalanx amputation	5
Hip disarticulation	2
Transfemoral amputation	28
Knee disarticulation	7
Transtibial amputation	32
Syme amputation	1
Chopart amputation	2
Metatarsal-phalanx amputation	2
Totally	96

We performed fasciotomy in only nine patients (Table 2). The mean time under rubble was 19.88 ± 19.99 h. The mean surgery time was 32.66 ± 22.55 h. Twenty-seven patients who had undergone fasciotomy were referred to our hospital. We evaluated 44 patients, including those operated by the plastic and reconstructive surgery department. Among 44 patients, two patients died, and three patients underwent amputation.

**Table 2:** Fasciotomies, surgical sites and outcomes

Patients	Surgical site	Time under the rubble (h)*	Operation time (h)*	Secondary surgery**
1	Left arm	6	8	–
2	Right forearm	1	8	–
3	Left leg	56	60	Debridement
4	Left leg	48	66	Transtibial amputation
5	Left leg	6	16	–
6	Right forearm	6	60	Debridement
7	Right forearm	18	22	–
8	Left leg	10	18	–
9	Left forearm	28	36	–

*h: hours, **except for reconstructive surgeries

Two hundred sixty-four patients had crush injuries. Patients with less than a day of hospitalization, patients referred to our hospital for the second week, and patients with CK concentration < 1000 U/L were excluded from the study. As a result, 233 patients with crush syndrome were included in the study. Age groups, gender, nationality, death, surgical procedure, affected limb part and number, trunk trauma, and dialysis requirement of the patients are summarized in Table 3.

**Table 3:** Clinical and demographic characteristics of patients with crush syndrome

	*n*	%
Age groups		
1–7	22	(9.4)
8–17	52	(22.3)
18–64	147	(63.1)
65+	12	(5.2)
Gender		
M	111	(47.6)
F	122	(52.4)
Nationality		
TR	182	(78.1)
SY	51	(21.9)
Status		
Alive	218	(93.6)
Exitus	15	(6.4)
Surgery		
(−)	75	(32.2)
After admission	69	(29.6)
Before admission	41	(17.6)
Both	48	(20.6)
Dialysis		
(−)	101	(43.3)
(+)	132	(56.7)
Amputation		
(−)	161	(69.1)
(+)	72	(30.9)
Fasciotomy		
(−)	192	(82.4)
(+)	41	(17.6)
Thigh		
None	113	(48.5)
Unilateral	67	(28.8)
Bilateral	53	(22.7)
Leg		
None	72	(30.9)
Unilateral	94	(40.3)
Bilateral	67	(28.8)
Foot		
None	72	(30.9)
Unilateral	97	(41.6)
Bilateral	64	(27.5)
Arm		
None	177	(76.0)
Unilateral	51	(21.9)
Bilateral	5	(2.1)
Forearm		
None	159	(68.2)
Unilateral	64	(27.5)
Bilateral	10	(4.3)
Hand		
None	160	(68.7)
Unilateral	64	(27.5)
Bilateral	9	(3.9)
Abdominal trauma		
(−)	204	(87.6)
(+)	29	(12.4)
Thoracic trauma		
(−)	186	(79.8)
(+)	47	(20.2)
Lower limbs side		
None	44	(18.9)
Unilateral	110	(47.2)
Bilateral	79	(33.9)
Injured lower limb parts (Thigh, Leg, Foot)		
0	44	(18.9)
1	30	(12.9)
2	62	(26.6)
3+	97	(41.6)
Upper limb side		
None	143	(61.4)
Unilateral	76	(32.6)
Bilateral	14	(6.0)
Injured upper limbs parts (Arm, Forearm, Hand)		
0	143	(61.4)
1	20	(8.6)
2	27	(11.6)
3+	43	(18.5)

**Fig. 2:**
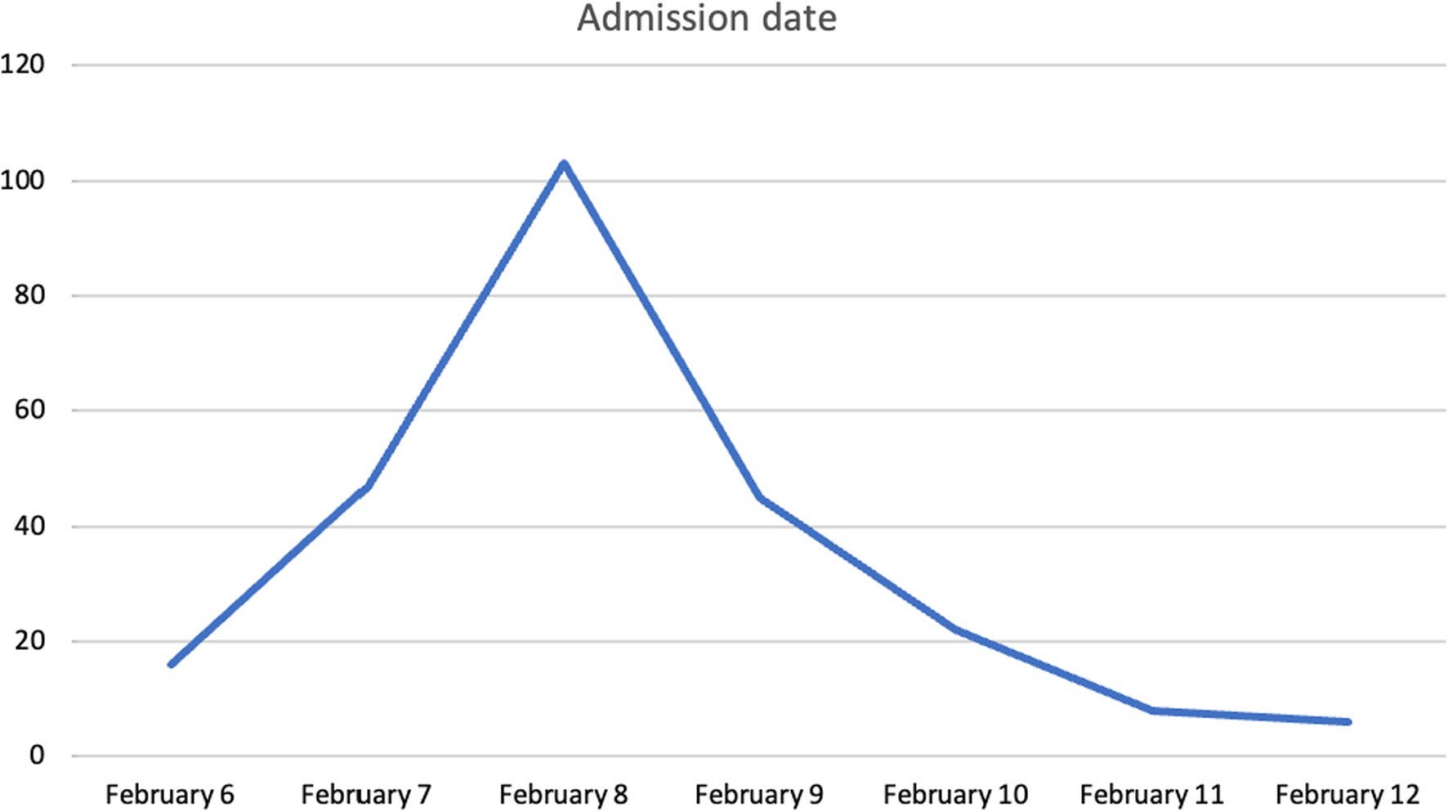
Distribution of patients' hospital admission dates

The mean time under the rubble was 41.89 ± 29.75 h. Most patient admissions were in the first hours of the third day (Fig. 2). Dialysis was performed at least once in 132 patients (56.65%). The mean peak CK concentration was 69.817.69 ± 134.812.04 U/L.

The correlation of parameters with mortality was examined. Lower extremity injury, abdominal trauma, and thoracic trauma were associated with mortality (Table 4). Of the 15 dead patients, 12 had at least one thigh injury. Three patients without thigh injury had abdominal trauma.

**Table 4:** Correlation of injured limb parts, surgical procedures and trunk injuries with mortality

	Status				*p*
	Alive		Exitus		
	*n*	%	*n*	%	
Amputation					
(−)	148	(91.9)	13	(8.1)	0.128
(+)	70	(97.2)	2	(2.8)	
Fasciotomy					
(−)	179	(93.2)	13	(6.8)	0.654
(+)	39	(95.1)	2	(4.9)	
Thigh					
(−)	110	(97.3)	3	(2.7)	0.033
Unilateral	62	(92.5)	5	(7.5)	
Bilateral	46	(86.8)	7	(13.2)	
Leg					
(−)	71	(98.6)	1	(1.4)	< 0.001
Unilateral	91	(96.8)	3	(3.2)	
Bilateral	56	(83.6)	11	(16.4)	
Foot					
(−)	72	(100.0)	0	(.0)	< 0.001
Unilateral	94	(96.9)	3	(3.1)	
Bilateral	52	(81.3)	12	(18.8)	
Arm					
(−)	165	(93.2)	12	(6.8)	0.350
Unilateral	49	(96.1)	2	(3.9)	
Bilateral	4	(80.0)	1	(20.0)	
Forearm					
(−)	149	(93.7)	10	(6.3)	0.896
Unilateral	60	(93.8)	4	(6.3)	
Bilateral	9	(90.0)	1	(10.0)	
Hand					
(−)	152	(95.0)	8	(5.0)	0.107
Unilateral	59	(92.2)	5	(7.8)	
Bilateral	7	(77.8)	2	(22.2)	
Abdominal trauma					
(−)	194	(95.1)	10	(4.9)	0.011
(+)	24	(82.8)	5	(17.2)	
Thoracic trauma					
(−)	177	(95.2)	9	(4.8)	0.048
(+)	41	(87.2)	6	(12.8)	

The correlation of the parameters to peak CK was examined. Peak CK concentration increased significantly with amputation, lower limb injury, abdominal trauma, and thoracic trauma (Table 5). There were no similar results for dialysis. There was no significant correlation between amputation (*p* = 0.076), fasciotomy (*p* = 0.937), thigh injury (*p* = 0.246), leg injury (*p* = 0.083), abdominal trauma (*p* = 0.303), thoracic trauma (*p* = 0.902), and dialysis.

**Table 5:** Correlation of injured limb parts, surgical procedures and trunk injuries with peak CK

	Mean	SD	Median	25th	75th	*p*
Status						
Alive	66,349.8	133,432.7	28,803.5	8779.0	58,758.0	0.161
Exitus	107,389.5	153,110.0	40,744.0	15,789.0	164,539.0	
Amputation						
(−)	59,705.5	125,919.5	24,800.0	7744.0	49,553.0	0.002
( +)	89,757.3	151,681.5	41,887.0	15,048.0	90,065.5	
Fasciotomy						
(−)	68,663.6	132,928.9	31,003.5	8726.0	63,001.0	0.777
(+)	70,529.2	144,936.1	26,999.0	11,937.0	42,285.0	
Thigh						
(−)	29,141.8	71,989.6	15,092.0	5050.0	33,697.0	< 0.001
Unilateral	74,273.4	113,576.1	38,470.0	13,615.0	93,183.0	
Bilateral	147,278.5	209,275.8	58,758.0	37,855.0	146,904.0	
Leg						
(−)	30,465.9	89,853.1	12,288.5	3590.5	27,045.5	< 0.001
Unilateral	50,457.4	85,569.7	30,116.5	8673.0	58,819.0	
Bilateral	136,396.6	195,309.0	56,259.0	35,828.0	147,290.0	
Foot						
(−)	47,370.6	118,996.1	19,451.0	5692.5	39,555.5	< 0.001
Unilateral	55,141.2	113,490.2	27,411.0	7698.0	52,258.0	
Bilateral	114,308.3	168,552.2	48,980.5	30,025.5	103,630.5	
Arm						
(−)	71,900.2	144,544.0	29,026.0	8779.0	58,758.0	0.897
Unilateral	59,054.0	99,820.3	36,317.0	9299.0	65,472.0	
Bilateral	67,402.4	91,447.8	29,824.0	11,937.0	56,259.0	
Forearm						
(−)	65,978.6	130,884.9	29,810.0	7774.0	58,758.0	0.301
Unilateral	67,456.5	134,998.4	27,457.0	9667.0	63,316.0	
Bilateral	126,729.5	190,207.2	45,092.5	29,824.0	182,277.0	
Hand						
(−)	66,246.8	127,191.7	29,817.0	8020.5	60,287.0	0.227
Unilateral	68,215.6	144,399.2	27,457.0	9667.0	55,143.5	
Bilateral	123,313.1	194,191.2	56,259.0	42,045.0	92,309.0	
Abdominal trauma						
(−)	63,105.2	130,747.7	27,542.0	8075.0	56,956.0	0.003
(+)	110,401.4	156,785.4	49,714.0	16,035.0	123,064.0	
Thoracic trauma						
(−)	61,683.3	128,814.2	27,447.5	8267.0	57,653.0	0.022
(+)	97,915.3	154,384.3	40,210.0	15,789.0	111,728.0	

**Fig. 3:**
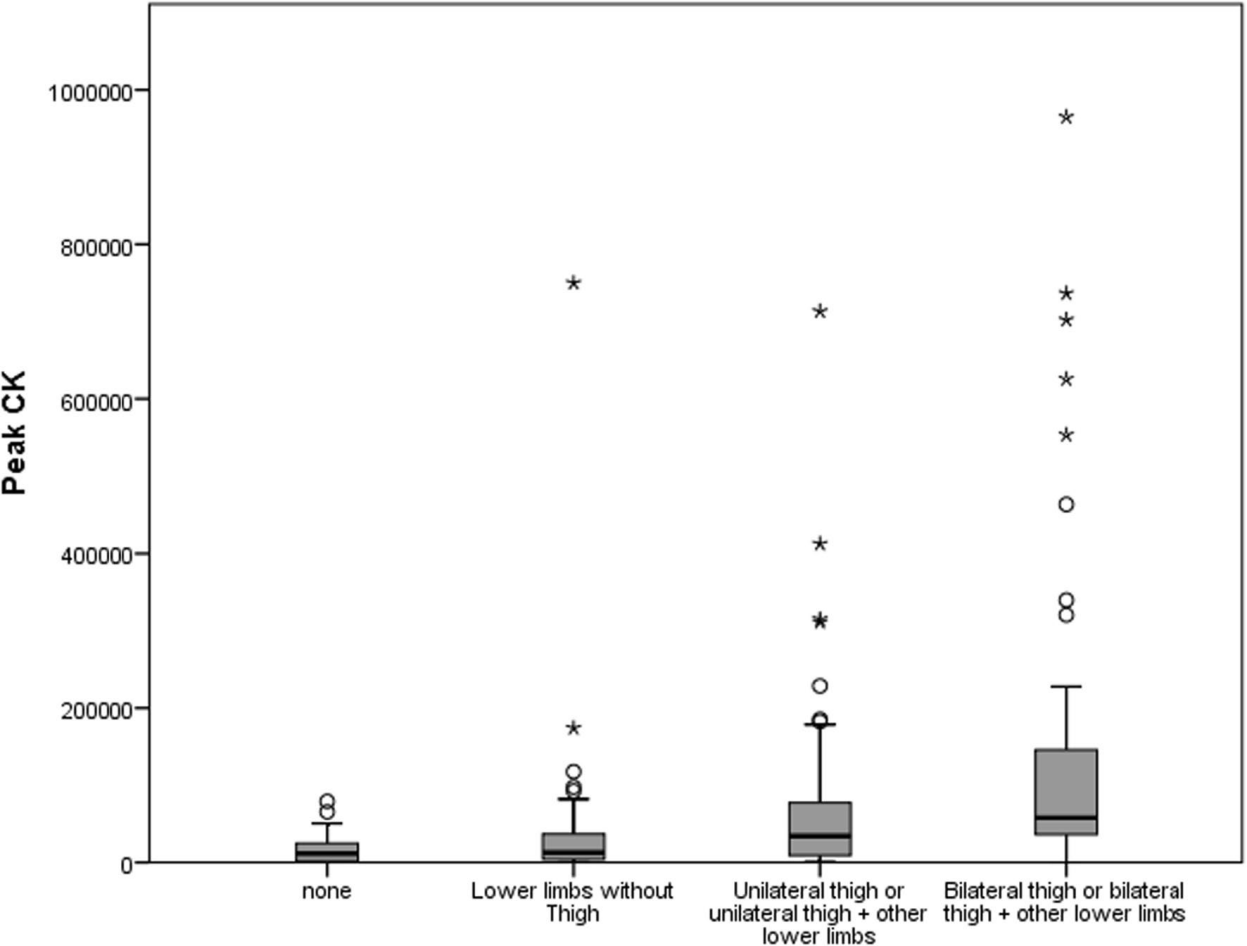
Patients divided into four groups according to the parts of the lower limb affected

CK concentration in Group 1 and Group 2 was significantly lower than in Group 3 and Group 4 (*p* < 0.001). CK concentration in Group 4 was significantly higher than in Group 3 (*p* = 0.012) (Fig. 3). Similarly, mortality rates in the same groups were 0.0%, 3.7%, 6.8%, and 12.7%, respectively (*p* = 0.028). Mortality was significantly increased in patients with thigh injuries. No significant correlation was found between the number of affected upper limb parts and mortality (*p* = 0.486).

No significant correlation was found between the time under the rubble and the peak CK. (*r* = − 0.012, *p* = 0.847). The median value for the trapped time in the group requiring dialysis was 40 (IQR: 16–48) hours, and this value was 36 (IQR: 15–56) hours in the group not requiring dialysis. Time under rubble was equivalent in those who needed and did not need dialysis (*p* = 0.847).

The time under the rubble was 48 h (IQR: 40–60) in patients who died and 36 (IQR 13–50) hours in survivors. (*p* = 0.055) No significant correlation was found.

## Discussion

The primary goal of treatment in crush injury should be to save lives, secondly, to preserve limb function. In patients with crush injury, severely traumatized limbs with tissue necrosis are a potential source of myoglobin and potassium release into the circulation. This muscle necrosis is also a source of infection, sepsis, and death [10]. Therefore, early amputations may save lives, and survival chances should not be compromised by desperate and inefficient attempts to save a limb [7]

The indication and timing of fasciotomy in crush injury are controversial. On the one hand, compartment syndrome is considered a surgical emergency in which fasciotomy is the only treatment because muscle necrosis can occur if the intracompartmental pressure rises more than 30 mm Hg for more than 8 h [11]. While successful results have been reported in fasciotomy performed before 6–12 h, which is called early fasciotomy, generally poor results have been reported in late fasciotomy [12, 13]. On the other hand, although compartment syndrome and crush syndrome are considered part of the same disease spectrum, they are different, and fasciotomy is not considered the first-line treatment for crush syndrome [11]. Because necrosis occurs in some muscles before any increase in compartment pressure occurs in crush injury, it is clear that fasciotomy applied after crush injury cannot heal muscle damage that has occurred in the same compartment, and the priority should be to preserve renal functions [14, 15]. The primary indication for fasciotomy in these patients is the injuries in which the pulse cannot be taken for a few hours, the muscle damage is minimal, and the compartment pressures increase slightly [16]. We performed fasciotomy on only nine patients since the patients had long times under the rubble and the admissions were late, and we followed up on many compartment syndromes nonsurgically.

Complications such as indications of fasciotomy should also be addressed. Sever et al. reported 24.8% sepsis and 16.4% death after fasciotomy. These rates were higher than those without fasciotomy [17]. Duman et al. reported amputation due to 25% intractable infection in patients who underwent fasciotomy [18]. Zhang et al. reported that 33.0% of amputations were due to incomplete or delayed fasciotomies and uncontrolled infection of fasciotomy wounds [19]. Günal et al. reported no sepsis, but 81% of patients developed wound infection after fasciotomy and were treated with debridement [20]. The general opinion is that fasciotomy is a high risk for infection. In addition, it has been reported that the infection sequelae are much worse than late muscle contracture that may result from muscle fibrosis and do not contribute to the functional recovery of the muscle in the long term [16, 21, 22].

Unlike previous studies, sepsis did not develop due to fasciotomy. Only three (20%) patients who underwent fasciotomy after 36 h were debrided or amputated due to muscle necrosis and mild infection, and no patient died. Two patients referred to our clinic after fasciotomy died without surgical intervention.

Fasciotomy performed on limbs with dead muscle tissue will increase reperfusion and metabolic products from necrotic tissue. This post-ischemic reperfusion is an effective mechanism in the development of crush syndrome [23]. Kantarci et al. reported that fasciotomy was the strongest indicator of dialysis requirement [6]. Sever et al. reported the dialysis requirement in 83.9% of those who underwent fasciotomy and 65.2% of those who did not [17]. In the study of Matsuoka et al., unlike other studies, the ratio of patients with and without fasciotomy requiring hemodialysis was similar [22]. In our study, fasciotomy was not significantly associated with dialysis requirement, peak CK concentration, and mortality. The lack of increase in the severity of crush syndrome and the low infection rate can be explained by the knowledge we have gained from past experiences and the low number of fasciotomies performed in patients with late admission, the correct indication, and appropriate wound care.

Amputation is the primary surgical management strategy for crush syndrome. It is a life-saving procedure but should be the last choice [22]. We performed amputation in 26% of patients with crush syndrome. These patients had a long time trapped under the rubble, had no circulation, or had severe muscle necrosis. In the study of Sever et al., 121 amputations were performed on 95 patients, 81.7% of whom were located in the lower extremities. The presence or absence of amputations did not differ significantly between dialysis and nondialysis victims [17]

In our study, the peak CK concentration was significantly higher in amputated victims, but there was no significant difference in dialysis need and mortality. In this heterogeneous patient population, the most critical patient group was those with indications for amputation. Despite high peak CK concentrations, low mortality rates and low dialysis requirements demonstrate the importance of amputations in crush syndrome.

Fifteen (6.4%) patients with crush syndrome died. At least one thigh was affected in 12 patients, and three victims without thigh injuries had severe abdominal injuries. Two patients had fasciotomy wound before admission. In the study of Oda et al., 50 patients (13.4%) with crush syndrome died, and an increase in mortality rate (50%) was reported in patients with abdominal injuries [24]. In the study of Sever et al., 29 (30.5%) patients who underwent amputation died, while 68 (12.5%) deaths were recorded in the remaining 544 patients. They also reported that thoracic and abdominal trauma was a significant mortality predictor [17]. The mortality rate was lower than in previous studies. The mortality rate was 2.8% in those who underwent amputation and 8.1% in those who were not, and there was no significant difference. Amputation was planned in 13 patients, and they died before amputation could be performed due to their systemic and metabolic conditions and chaos. Similar to previous studies, we found a significantly higher mortality rate in patients with abdominal and thoracic injuries.

Demirkıran et al. reported lower limb injuries in 16 patients and upper limb injuries in four [25]. Oda et al. reported that the injury was predominantly in the lower limbs (274 patients, 73.7%), followed by the upper limbs (36 patients, 9.7%) and the trunk (32 patients, 8.6%). He suggested that the crushed limb numbers and peak CK concentration provide a practical and rapid estimation of crush syndrome severity [24]. Sever et al. reported a significant correlation between the number of limbs and the dialysis requirement [17]. The clinical status worsens as the number of affected extremities increases, which is understandable given the muscle mass increase. In our clinical observation, we noticed that the clinical status deteriorated rapidly in patients with thigh injuries. The poor prognosis of thigh injury may also be explained by increased muscle mass, but it has not been reported before. We found a significant increase in CK concentration and mortality with the increase in the number of affected parts of the lower limbs. Especially in bilateral thigh injuries, the mortality rate was approximately 13%. But there was no significant correlation with dialysis. This can be explained by the decrease in the dialysis requirement when amputation is performed. There was no significant increase in CK concentration and mortality in patients with upper limb injuries. In light of our experience and study results, we recommend that quick and radical amputation decisions be made in patients with pulseless, cyanotic, and severely crushed lower limb and thigh injuries. In upper extremity crush injury, monitoring of metabolic status and extremity-sparing methods can be preferred, and future reconstructive interventions can be given a chance.

The time under the rubble may not reflect the severity of the injury or potential medical complications. Renal and cardiac complications are more sensitive to the magnitude of the pressure and the mass of the crushed muscle groups [26]. In the study by Shimazu et al., the mean time under rubble was 6.7 h [11]. In the study of Matsuoka et al., time under rubble was 7.0 ± 3.3 h [22]. In the study of Demirkıran et al., the mean time from earthquake to rescue was 24.10 h [25]. In the study of Duman et al., the mean admission time was 15.75 h in patients who underwent amputation and 10.5 h in those who did not require amputation [18]. Oda et al. reported the mean time under rubble was 9.0 ± 13.0 h. There was no correlation between the trapped time and the severity or prognosis of crush syndrome [24]. In our study, the time under rubble was much longer than previously reported (41.89 ± 29.75 h). However, no significant correlation was found between the time under the rubble and CK concentration, dialysis requirement, or mortality.

### Limitations

Retrospective data collection has several disadvantages because many of the parameters to be evaluated may not be recorded. Especially in such disasters, patient records may be insufficient due to the chaos. In this single-center study, our patient records were sufficient. In addition, surgical methods and indications were heterogeneous. As in all previous studies, we did not consider the gluteal muscles, lumbar region muscles, and muscle compartments around the shoulder. Crushing of these muscles in abdominal and thoracic trauma may affect the clinical status. On the other hand, despite these disadvantages, we hope clinical findings observed and analyzed in this study can provide beneficial messages for saving lives and making the correct surgical decision in future disasters.

## Conclusion

The treatment approach to crushed limb injuries caused by earthquakes is not similar to compartment syndrome. In delayed cases, the patient's life should not be risked by fasciotomy. Considering the increase in mortality and worsening of clinical status in thigh and multiple lower extremity injuries, prompt and radical surgical decisions should be made. Since peak CK concentration, mortality, and dialysis requirement do not increase in upper extremity injuries, amputation should not be rushed, and extremity-sparing procedures should be evaluated considering the clinical status.

## Data Availability

The data that support the findings of this study are available from the corresponding author, [B.K.], upon request.

## Abbreviations

CK: Creatinine kinase
U/L: Unite per liter
IQR: Interquartile range
