# Correction: 6 February 2023, orthopedic experience in Kahramanmaraş earthquake and surgical decision in patients with crush syndrome

**DOI:** 10.1186/s13018-024-04626-x

**Published:** 2024-02-19

**Authors:** Bugra Kundakci, Akif Mirioglu, Mustafa Tekin, Melih Bagir, Omer Sunkar Bicer, Yusuf Kemal Arslan, Cenk Ozkan, Hilmi Serdar Ozbarlas

**Affiliations:** 1 Department of Orthopaedics and Traumatology, Cukurova University Faculty of Medicine, Adana, Turkey; 2 Department of Biostatistics, Cukurova University Faculty of Medicine, Adana, Turkey

**Correction: Journal of Orthopaedic Surgery and Research (2013) 18:537** 10.1186/s13018-023-04001-2

In this article ref. 23 Epstein FH, Odeh M. The role of reperfusion-ınduced ınjury in the pathogenesis of the crush syndrome. New Engl J Med. 1991;324:1417–22. 10.1056/nejm199105163242007 was incorrect and should have been

Odeh M. The role of reperfusion-induced injury in the pathogenesis of the crush syndrome. New Engl J Med. 1991;324:1417–22. 10.1056/nejm199105163242007.

The original article has been revised.

## References

[CR1] Kundakci B, Mirioglu A, Tekin M (2023). 6 February 2023, orthopedic experience in Kahramanmaraş earthquake and surgical decision in patients with crush syndrome. J Orthop Surg Res.

